# Functional consequences of mutations in the *Drosophila* histamine receptor HCLB

**DOI:** 10.1016/j.jinsphys.2009.08.016

**Published:** 2010-01

**Authors:** Shazie Yusein, Adrian Wolstenholme, Eugene Semenov

**Affiliations:** a Institute of Molecular Biology, Department of Molecular Neurobiology, Akad. G. Bonchev bl.21, Sofia 1113, Bulgaria; b University of Bath, Department of Biology and Biochemistry, Bath BA2 7AY, UK

**Keywords:** Histamine receptor, Anaesthesia, Avermectin, Visual signalling

## Abstract

The gene *hclB* encodes a histamine-gated chloride channel subunit in *Drosophila melanogaster*. Mutations in *hclB* lead to defects in the visual system and altered sensitivity to the action of ivermectin. To investigate whether this member of the Cys-loop receptors is common across the Insecta, we analysed the genomes of seven other insect species (Diptera, Hymenoptera, Coleoptera) and revealed orthologues of *hclB* in all of them. Sequence comparisons showed high identity levels between the orthologues, indicating similar constraints and conserved function between the species. Two *D. melanogaster* mutants, *hclB*^*T1*^ (P293S) and *hclB*^*T2*^ (W111*, a null mutation) were tested for the lapse into, and recovery from, paralysis induced by high temperature or the anaesthetic action of halothane. At 41 °C, the *hclB*^*T2*^ flies lapsed into coma faster than wild-type or the *hclB*^*T1*^ flies, while both mutants recovered more slowly. A substantially impaired recovery rate was also observed in *hclB*^*T1*^ after anaesthesia with halothane. Enhanced synaptic signalling at low-intensity light stimuli was registered on electroretinograms recorded from the two mutant strains. Our results suggest that HCLB may play an essential and conserved role in insect neurophysiology.

## 1 Introduction

Fast neurotransmission mediated by histamine (Hardie, 1987, 1989a) is a common signalling mechanism in arthropod visual pathways (Hardie, 1989b; Nassel, 1999; Stuart, 1999; Stuart et al., 2007). Two genes, *hclA* and *hclB* (the genetic nomenclature is according to Geng et al., 2002), each encoding a distinct ionotropic histamine receptor subunit, were recently identified in *Drosophila* (Geng et al., 2002; Gisselmann et al., 2002; Witte et al., 2002; Zheng et al., 2002). *In vitro* expression studies demonstrated the formation of homomeric HCLA and HCLB chloride channels (Gisselmann et al., 2002; Zheng et al., 2002), or heteromeric HCLA/HCLB channels (Zheng et al., 2002), all of which responded to histamine. Heterologously expressed HCLB and HCLA/HCLB channels are also been activated by the macrocyclic lactone, ivermectin (Zheng et al., 2002), whose mode of action is the specific and essentially irreversible activation of ligand-gated chloride channels. The two subunits have the highest sequence identity (30–40%) to mammalian glycine and GABA receptors.

In the visual system, HCLA is expressed in the large monopolar cells (LMCs), while HCLB is exclusively localized to the glial cells in the lamina (Pantazis et al., 2008). *hclA* (initially known as *ort*) mutations lead to defective vision, documented as electroretinogram (ERG) records lacking the on- and off-transient components (Koenig and Merriam, 1977; O’Tousa et al., 1989). The studies of Heisenberg (1971) and Coombe (1986) demonstrated that the lack of these transient components is the result of impaired synaptic transmission between the photoreceptors and their targets, the large monopolar cells in the lamina, which is consistent with the expression data. By contrast, ERG records from *hclB* mutants have been reported to contain both components; the on-transients only (Pantazis et al., 2008) or both on- and off-transients (Yusein et al., 2008) having higher amplitudes than those from control flies. At the same time, the LMCs response to low-intensity brief flashes, where the on- and off-components are not separated, were shown to be less sensitive than the corresponding responses of the controls (Pantazis et al., 2008). It was suggested that the HCLB channels participate in the modulation of the visual responses. However, it remains unknown if the sensitivity of on- and off-transient responses in all *hclB* mutants is affected in a similar way.

The use of the reporter gene strategy (Hong et al., 2006) allowed the mapping the expression of these proteins in many other cells of adult brain and the thoracic ganglia. It is well-known that mutations in a single synaptic protein may result in diverse neurological effects, as reported for the *Drosophila* voltage-sensitive sodium channel *paralytic* (Loughney et al., 1989), where mutations result in hypersensitivity to increased temperature and defects in olfaction, circadian rhythms and courtship (reviewed in: Wu and Ganetzky, 1992; Smith, 1996; Young, 1998; Greenspan and Ferveur, 2000). Known non-visual phenotypes of the *ort* mutant flies include abnormal responses to mechanical shock (bang sensitivity) or diethyl ether (Iovchev et al., 2002), and mutant-specific temperature preferences (Hong et al., 2006), all implying that HCLA has functions outside the visual system. Hong et al. (2006) also described the first *hclB* null mutant (*HisCl1*^*134*^) and demonstrated that it not only prefers a higher temperature than normal flies, but also has a reduced tolerance for high temperatures. Recently, we identified two new *Drosophila* mutants, *hclB*^*T1*^(P293S) and *hclB*^*T2*^(W111*, a null mutation), and showed that they have allele-specific visual system phenotypes and altered susceptibility to ivermectin (Yusein et al., 2008). Since the proline residue at position 293 affected in *hclB*^*T1*^ is highly conserved across the ligand-gated chloride channel family, we explore here the effect of its substitution on the tolerance of flies to high temperature. As it is known that the HCLA subunit is involved in the response to anaesthesia (Iovchev et al., 2002), we also explored the influence of HCLB-containing channels on sensitivity to the anaesthetic agent, halothane.

We show that mutations in *hclB* lead to allele-specific responses of mutant flies to both high temperature and anaesthesia. We also demonstrate that the absolute sensitivity of both on- and off-transient responses in the ERG is increased to almost the same degree in the *hclB* mutants. The high degree of identity of HCLB orthologues from various insect species allows us to conclude that the gene has an important role in nervous systems across the Insecta.

## 2 Materials and methods

### 2.1 Drosophila stocks

Two strains with mutant *hclB* alleles having the genotype: *st hclB*^*T1*^*/TM3*, *Sb* and *st hclB*^*T2*^*/TM3*, *Sb* were described in Yusein et al. (2008). The flies were kept on yeast-molasses medium at 25 °C. For all experiments we used 3–7-day-old hemizygous females produced by crossing of mutant strains with flies *Df(3R)E79/MRS*, *Sb* where the deficiency (86F1–87B9) eliminates the *hclB* chromosomal region. Hemizygous control flies were obtained in a similar way from Oregon R (OR).

### 2.2 Genetic nomenclature

Here we follow the nomenclature proposed by Geng et al. (2002): *hclA* (*ort*) and *hclB*. The two genes are also referred to as *HisCl2* and *HisCl1* respectively by Zheng et al. (2002) and *HisCl*-*α1* and *HisCl*-α2 by Gisselmann et al. (2002).

### 2.3 Bioinformatics analysis

The *hclB* orthologues from *Apis mellifera* (honeybee, Hymenoptera), *Anopheles gambiae* (malaria mosquito, Diptera), *Aedes aegypti* (the yellow fever mosquito, Diptera), *Tribolium* *castaneum* (red flour beetle, Coleoptera), *Drosophila* *ananassae*, *Drosophila* *pseudoobscura*, and *Drosophila* *virilis* were identified under the analysis of alignments of the *Dm*-*hclB* (NM_169429) sequence to the corresponding whole genome sequences from NCBI trace archives at (http://www.ncbi.nlm.nih.gov/BLAST/tracemb.shtml). Alignments of the predicted amino-acid sequences to known protein database were performed by the use of the Predict Protein software (http://www.cubic.bioc.columbia.edu/predictprotein/). The HCLBs from *Drosophilidae* species were aligned and compared by whole protein sequences (1–426aa of Dm-HCLB), whereas other *Insecta* species were aligned as following Aeae: 35–426aa; Anga: 29–426aa; Amel: 51–371aa; Tric: 51–426aa because of incomplete genome sequences.

### 2.4 Behavioral assays

#### 2.4.1 Knock-down analysis

Flies were placed in groups of ten in the vials, 2 cm in diameter, with cotton plugs by brief anaesthesia with carbon dioxide. After 1 h recovery they were put into an incubator with a transparent front, set at 41 °C. The number of flies that were unable to stand and fell to the bottom of vials was recorded at 1 min intervals. After 10 min all flies were knocked down.

#### 2.4.2 Measurement of arousal time after heat shock

The assays were modified from Hong et al. (2006). After knock-down analysis, flies were immediately removed from the thermostat and placed in plastic Petri dishes at 20 °C. The number of flies that could stand and walk was counted; for the first 30 min the number of aroused flies was recorded at 5 min intervals. All recovered animals were removed from the Petri dish by aspiration with a pipette to avoid any influence on the rest. After measurements at 45 and 60 min flies were replaced in vials containing a minimal medium covered with living yeasts and their recovery was measured after 3 and 24 h.

#### 2.4.3 Measurement of time for anaesthesia with halothane

All experiments were performed with halothane (‘Narcotan’, Zentiva International, Czech Republic) in a temperature controlled room at 22 °C. Adults from the three genotypes were anaesthetized in a desiccator of 5400 cm^3^ volume. The surfaces of parts that can detach were coated with silicone. An oval-shaped easel with places for vials was placed into the desiccator. A glass dish was placed into the middle of the easel and liquid anaesthetic was poured from the hole of the lid. Ten flies were placed in each vial by brief anaesthesia with carbon dioxide. Flies were allowed to recover for 1 h. Then they were placed into the desiccator. The recording started immediately after closing of the lid and addition of 5 ml halothane. During the experiment a saturated atmosphere of halothane was gradually reached. Every minute flies that were unable to stand and fell to the bottom of vials were counted. All flies were paralyzed after 15 min.

#### 2.4.4 Time of recovery after anaesthesia with halothane

The flies anaesthetized as described above were replaced into plastic Petri dishes at 25 °C. The recording of fly recovery was performed in the same way as the arousal time after heat shock. The viability of flies was counted after 24 h.

### 2.5 ERG recording

Recordings were carried out as described by Yusein et al. (2008). The electroretinograms were recorded using glass pipette microelectrodes with a tip diameter of 15–20 μm. The microelectrodes were filled with Ringer solution (in mmol/l: NaCl 130, KCl 4.7, CaCl_2_ 1.9, MgCl_2_ 4, HEPES 1.3; pH 7.14). The ERG responses were amplified at a bandpass of 0–1000 Hz using low noise WPI ISO-DAM preamplifier. They were digitized at 5 kHz and analysed using WPI LAB-Trax4 Data acquisition system (Data-Trax software). Diffuse light from green LUXEON^®^V LED (LXHL-PMo2; Lumileds Future Electronics) with a dominant wavelength of 530 nm was used for light stimulation. The stimulus intensity was changed at 0.5 log unit steps within a range of 5 log units. The maximal intensity used (denoted by 0) was 5.39 × 10^6^ quanta s^−1^ μm^−2^ at the plane of the eye. After 2 min dark adaptation, intermittent stimuli with 2 s ON and 8 s OFF periods were given.

### 2.6 Data analysis

Student's *t*-test was used for statistical evaluation of all data.

## 3 Results

### 3.1 Sequences variations in HCLB among different taxonomic groups of Insecta

**Fig. 1:**
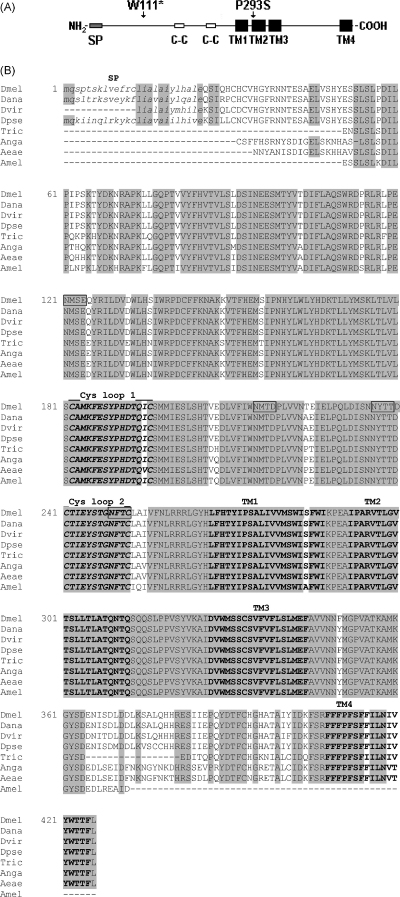
(A) Schematic map of the structural domains in HCLB with the position of the amino-acids affected in two mutants *hclB*^*T1*^ (P293S) and *hclB*^*T2*^ (W111*). (B) Alignment of HCLB sequences from *D. melanogaster* (Dmel), *D.ananassae* (Dana), *D. virilis* (Dvir), *D.pseudoobscura* (Dpse), *T. castaneum* (Tric), *A. gambiae* (Anga), *A. aegypti* (Aeae) and *A. mellifera* (Amel). Predicted signal peptides are italicised and indicated by SP, membrane-spanning regions (TMs1–4) are highlighted and the two Cys-loops (formed between C182–C196 and C241–252, respectively) are highlighted and italicised. Conserved amino-acids are boxed in gray, and the conserved N-linked glycosylation sites are boxed.

Histamine-gated chloride channels encoded by *hclA* and *hclB* in *Drosophila* are members of the Cys-loop superfamily of neurotransmitter-gated receptors. The HCLB subunit has a structure typical of this family: an N-terminal extracellular domain with two cysteine loops; four membrane-spanning domains (TM1–4); large intracellular loop and C-terminal domain (Fig. 1A). To reveal the genus-specific and interspecific polymorphisms we identified and analysed HCLBs from the genomes of three *Drosophilidae* and four non-*Drosophilidae* species (see Section 2). Fig. 1B shows the sequence conservation among seven HCLBs studied, when compared to the Dm-HCLB sequence. The amino-acid sequence identity between the HCLBs (vs. Dm-HCLB) in *Drosophilidae* species was more than 92%. The identity among HCLBs from other compared insect species and Dm-HCLB was also high: 86% for *A. aegypti*, 87% for *A. gambiae*, 92% for *A. mellifera* and 90% for *T. castaneum*. Four consensus sequences for N-glycosylation were conserved between all species. The genus-specific variations were limited mainly to the signal peptide and the intracellular domain sequence. Sequence alignments among all species revealed a pattern of evolutionary changes: highly identity in the N-terminal receptor domain and TMs1–4, with a more variable intracellular domain. The roughly 62aa long intracellular loop domain is the most variable part of the protein, with amino-acid identities ranging from 90 to 98% between drosophilid species to 63% between *Drosophila* *melanogaster* and other insects.

### 3.2 Altered tolerance for high temperature in hclB mutants

**Fig. 2:**
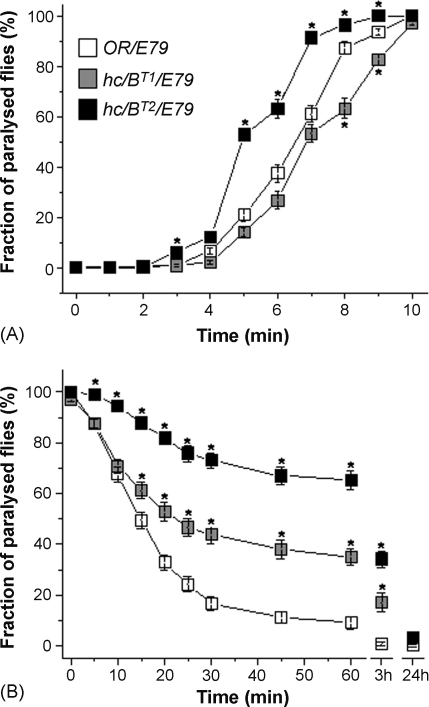
Dynamics of heat shock knock-down (41 °C) (A) and recovery at 20 °C (B) in *hclB* mutants (*hclB*^*T1*^*/Df(3R)E79* and *hclB*^*T2*^*/Df(3R)E79*) and control (*OR/Df(3R)E79*) flies. Asterisks indicate significant differences between the control and mutant flies with *p* < 0.001. Error bars represent SEM. Deficient chromosomes flies *Df(3R)E79* are designated as *E79*. 500 flies from each genotype were used.

The three test genotypes had very similar genetic background, produced by crossing of strains with flies *Df(3R)E79/MRS*, *Sb*. Only hemizygotes from the first progeny were used for the experiments. When tested for their ability to tolerate elevated temperatures, the null mutant flies, *hclB*^*T2*^, were knocked-down faster than control flies, as noted by Hong et al. (2006). Interestingly, the other mutant, *hclB*^*T1*^, had a phenotype similar to *OR/Df(3R)E79* except, possibly, after 8 and 9 min (Fig. 2A).

We then examined the recovery time after heat shock. We observed a notably impeded recovery in both *hclB* mutants, and this effect was greater in *hclB*^*T2*^ (Fig. 2B). Only 30% of *hclB*^*T2*^ flies were able to wake up within 1 h of recovery time, compared to 60% of *hclB*^*T1*^ and 90% of control flies. After 3 h all the control flies were recovered but not the *hclB* mutants. We measured the viability of unaroused flies after 24 h to determine whether heat treatment was lethal for them. As shown in Fig. 2B all the flies had recovered after this period of time.

### 3.3 Anaesthetic phenotype of the hclB mutants

**Fig. 3:**
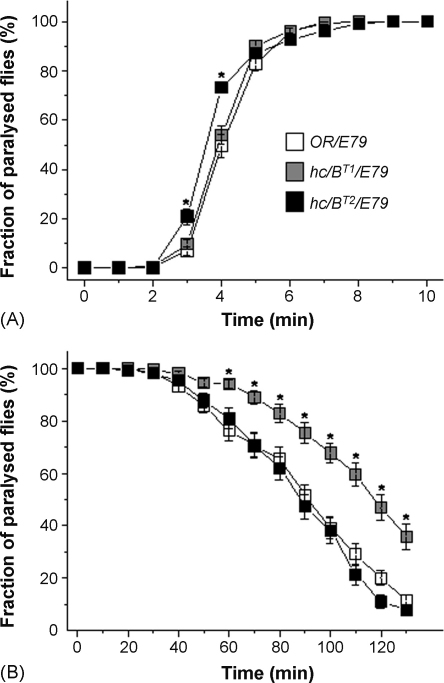
Lapse into (A), and recovery (B) from, anaesthesia with halothane in the three genotypes. Asterisks indicate significant differences between the control and mutant flies with *p* < 0.001. Error bars represent SEM. Deficient chromosomes flies *Df(3R)E79* are designated as *E79*. 220 flies from each genotype were used.

In our study we used loss of the ability to stand as the anaesthesia endpoint in *Drosophila*. Initially we measured the lethality of halothane. The flies were anaesthetized with the same amount (5 ml) of halothane for different periods of time and the lethality was scored after 24 h. No significant differences were obtained between three genotypes (data not shown); after exposure for 15 min all the flies were alive 24 h later, but exposure for 30 min resulted in 100% lethality. We therefore chose a non-lethal 15 min treatment period to compare the responses of *hclB* mutants and control flies to halothane. The null mutants, *hclB*^*T2*^, lost their postural control faster than *hclB*^*T1*^ and control flies (Fig. 3A). Their half knock-down times (KT_50_) were also different – for wild-type flies KT_50_ = 4.00 ± 0.08 min while for the *hclB*^*T2*^ KT_50_ = 3.47 ± 0.06 min (*p* < 0.001). The KT_50_ of *hclB*^*T1*^ mutants was 3.93 ± 0.07 min, which was not significantly different from the controls. The flies from all genotypes were lying on the bottom of vial after 8 min and were completely immobile up to 15 min. We also measured the time of recovery from anaesthesia with halothane. After 15 min anaesthesia the flies were replaced at 25 °C to recover. In contrast to the increased sensitivity to halothane shown in the onset of paralysis, *hclB*^*T2*^ mutants showed no changes from control flies in the time of recovery. However *hclB*^*T1*^ mutants had a significantly delayed recovery. After 2 h only half of *hclB*^*T1*^ flies were restored in comparison with more than 80% of control and *hclB*^*T2*^ mutants (Fig. 3B). The average body weight of control, *hclB*^*T1*^ and *hclB*^*T2*^ flies were very similar: 1.40, 1.34 and 1.35 mg/fly, respectively.

### 3.4 Effect of mutations in hclB on visual responses of Drosophila

**Fig. 4:**
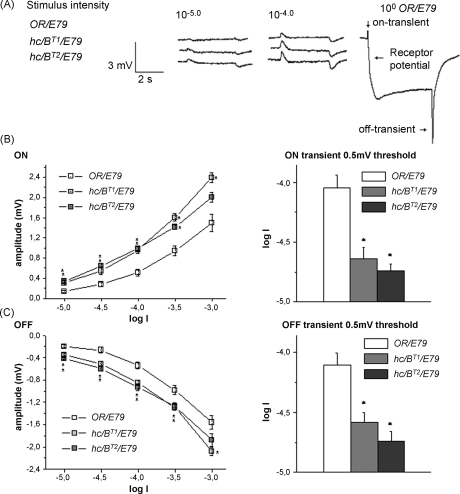
Representative ERGs from *hclB* mutants (10^−5^ and 10^−4^) and control flies *OR/E79* (10^−5^, 10^−4^ and 10^0^). Typical ERG contains sustained receptor potential and two transient components: on and off (A). Intensity-response (*V*/log *I*) functions (B, C left) and 0.5 mV thresholds (B, C right) of on- and off-transients in three genotypes was shown. The light intensity units are given as log *I*s. Error bars represent SEM. Seven flies from each genotype were used. Asterisks indicate significant differences between the control and mutant flies with *p* < 0.01.

The ERG of the *Drosophila* compound eye has a complex waveform and consists of contributions from receptor cells and the second-order cells in the lamina. The corneal-positive on-transient and the negative off-transient arise in the lamina and most of the sustained negative component reflects the depolarization of receptor cells (Fig. 4Aright) (Heisenberg, 1971; Coombe, 1986). As the thresholds of the ERG on- and off-transients are lower than the threshold of the photoreceptor component, only transient responses (“pure” laminar responses) could be obtained using stimuli of very low intensity (Heisenberg, 1971). We studied the intensity-response (*V*/log *I*) functions of the ERG on- and off-transients in a range of low stimulus intensities, including the range which was below the photoreceptor component threshold. From the *V*/log *I* functions, the 0.5 mV thresholds of the two ERG responses were derived for sensitivity assessment.

Original representative ERG records of a control fly as well as of the two *hclB* mutants are shown in Fig. 4A. The amplitudes of the on- and off-transients of the mutants were greater than the wild-type flies. In Fig. 4 the intensity-response curves and 0.5 mV thresholds of the ERG on-transients (Fig. 4B) and off-transients (Fig. 4C) are presented. It is clearly seen that the curves of the two mutants are shifted to the left along the stimulus intensity scale and that the 0.5 mV thresholds of both on- and off-transients of the mutants are lower than the threshold of the wild-type flies. This indicated a higher absolute sensitivity of both transients in the *hclB* mutants. The sensitivity of on- and off-responses was increased to a similar degree. There was no significant difference in the absolute sensitivity of the transient responses between the two *hclB* mutants. In no point along the stimulus intensity scale could we obtain a lower sensitivity of the responses from *hclB* mutant flies, as was reported by Pantazis et al. (2008) for LMCs from the *hisCl1*^*134*^ null mutant.

## 4 Discussion

When HCLBs from Insecta species were compared with *Dm-HCLB*, substitutions at several amino-acid residues were found in the sequences of their N-terminal domains, mainly concentrated near to the signal peptide. The high level identity between the N-terminal domains and the near to hundred percent identity of the membrane-spanning regions of all the compared insects indicate their significance for HCLB function. The TM2 is important in forming of the channel gate (Saul et al., 1999), in controlling ionic selectivity (Corringer et al., 1999; Carland et al., 2004), and in forming the binding sites for agents such as insecticides (Ffrench-Constant et al., 1993) and anaesthetics (Lobo et al., 2004; Jenkins et al., 2001). In *hclB*^*T1*^ mutant flies the P293S substitution affects a highly conserved amino-acid in this domain, leading us to predict that in this mutant HCLB*-*containing channels have an altered function. All sites for N-glycosylation are completely conserved across all the species we examined. The importance of N-linked glycosylation was shown by the finding that glycosylation of glycine receptor α1-subunits is a necessary prerequisite for homomeric receptor assembly and that receptor assembly is required for transit from the endoplasmic reticulum to the Golgi apparatus and subsequently to the cell membrane (Griffon et al., 1999). Several replacements were also found in the large intracellular loop. The intracellular loop between TM3–4 of 5-HT_3_ receptors has been determined to act as a downstream filter controlling the ion flow (conductance) into the cytoplasm. Charged residues framing these ion portals appear to determine the efficiency with which ions are transported (Kelley et al., 2003). The high degree of conservation of protein sequences observed across Insecta species for both HCLA (Iovchev et al., 2006) and HCLB (observed in this study) indicate that both have an equally important and conserved function.

Histaminergic mutants have a changed tolerance to high temperature. They were knocked-down faster and recovered from heat shock paralysis much slower compared with the control (Hong et al., 2006). These authors concluded that the defects in the histamine-signalling genes can cause low tolerance to high temperature or lower the upper thermal limit and that the fine regulation of temperature preferences could be controlled by circadian clock neurons where they found *hclB* expression. We independently confirm their results for a null mutant of *hclB* (with *hclB*^*T2*^) and also provide new information by examination of *hclB*^*T1*^ (P293S). It was shown that proline residue in this position is important both for charge selectivity (Corringer et al., 1999) and desensitization (Saul et al., 1999) of the channel. Proline is known to facilitate conformational changes via *cis*–*trans* isomerization of the peptide bond and to disrupt secondary structure motifs. By topological analysis it was predicted that an amino-acid substitution P250T in human glycine receptor α1 subunit leads to the loss of an angular polypeptide structure, thereby destabilizing open channel conformations (Saul et al., 1999). The P293S mutation in *hclB*^*T1*^ is in the equivalent position to the P250T mutation in the human glycine receptor. All these observations suggest that HCLB-containing channels in *hclB*^*T1*^ flies could have an altered function.

We observed an intermediate phenotype of *hclB*^*T1*^ flies in the recovery after heat shock experiments – the flies recovered slower than control but faster than *hclB*^*T2*^ suggesting that the allele might be hypomorphic. Interestingly, the *hclB*^*T1*^ mutants responded to heat shock in a similar way to control flies in the knock-down analysis. It might be that the function of HCLB in *hclB*^*T1*^ is sufficient for knock-down like control flies but inadequate for recovery, and this might be influenced by the composition of the channels involved in these neuronal circuits, whether they are homomeric or heteromeric (HCLB/HCLA).

We also studied the influence of the HCLB-containing channels on the circuits involved in anaesthesia and recovery after treatment with halothane. Heat- and anaesthesia-induced paralysis seemed very similar: the *hclB*^*T2*^ null mutants fell fast while the response of *hclB*^*T1*^ flies was similar to controls. A possible explanation is that these responses are due to one common neuronal circuit, because of the similar endpoints used in two experiments. However, the recovery of mutants after two treatments was different. *hclB* null mutants (which possess only HCLA channels) have a wild-type time of recovery after anaesthesia with halothane unlike *hclB*^*T1*^ mutants that have prolonged recovery. On other hand, null mutants in *hclA*, which have only HCLB channels, show a substantially prolonged recovery after treatment with diethyl ether (Iovchev et al., 2002). These findings suggest that HCLB-containing channels are candidate components of the *Drosophila* response to volatile anaesthetics. However it needs further investigations to determinate whether they are direct targets for anaesthetics, like some other members of the Cys-loop superfamily, or whether they modulate anaesthesia by other, indirect, mechanisms. Certain amino-acids in TMs1–3 of the glycine receptor are critical for anaesthetics effects and some of them are accessible only at open state of channel. The mechanism by which anaesthetics act on the receptor could be through occupation of the anaesthetic binding cavity formed by their membrane-spanning domains, preventing the closing of the channel (Lobo et al., 2004). A similar cavity has been found in GABA_A_ receptors (Jenkins et al., 2001). The observed altered anaesthetic phenotype in *hclB*^*T1*^ mutants are of interest for further investigations in view of the fact that the P293S substitution could affect the potential anaesthetic binding cavity in HCLB-contained channels.

The visual responses of several *hclB* mutants have been studied so far: two null mutants *hisCl1*^*134*^ (Pantazis et al., 2008) and *hclB*^*T2*^, as well as *hclB*^*T1*^ (Yusein et al., 2008). In the present study we demonstrated that the absolute sensitivity of both on- and off-transients, estimated by their 0.5 mV thresholds, was increased to almost the same degree. This is in agreement with the results obtained in our previous study (Yusein et al., 2008). In no point along the stimulus intensity scale could we obtain a lower sensitivity of the mutant responses, as reported by Pantazis et al. (2008) for the null mutant *hisCl1*^*134*^. A possible explanation of this discrepancy might a difference in the *hisCl1*^*134*^ flies background. In our study flies with wild-type eye colour only were used, while *hisCl1*^*134*^ are actually double mutant *white*^*1118*^; *hisCl1*^*134*^. More recently it has been reported that *white* mutants have about half the wild-type amount of histamine in the head (Borycz et al., 2008). Furthermore, the *white* protein is expressed in lamina epithelial glia, at the same place where Pantazis et al. (2008) found HCLB. Borycz et al. (2008) propose that histamine uptake by the epithelial glia might be *white* dependent. A genetic interaction between *white* and *hclB* therefore seems possible. In our preliminary studies on double mutants *st hclB*, the mutation in *scarlet*, known to be a binding partner of *white*, altered the effect of the *hclB* mutation on the on-transient amplitude (unpublished data). In spite of the discrepancies mentioned, the results of this study as well as of the previous studies, show that HCLB-mediated influences are involved in the sensitivity control of the visual responses, which may be important for keeping the responses out of saturation. As the HCLB channels have been immunolabeled exclusively in the lamina glial cells (Pantazis et al., 2008), the contribution of the glial cells to the sensitivity control seems to be important. We can only speculate if the glial cells exert their effects through neurotransmitter clearance, changes in the resistance (current distribution) or some other effects, dependent on the involvement of the glial cells in the lamina cell circuitry.
